# Time to steroid treatment in severe acute optic neuritis

**DOI:** 10.1002/brb3.1032

**Published:** 2018-06-22

**Authors:** Gro Helen Dale, Thor Petersen, Kristina Bacher Svendsen, Tove Christensen, Gunnar Houen, Toke Bek

**Affiliations:** ^1^ Department of Neurology Aarhus University Hospital Aarhus Denmark; ^2^ Department of Biomedicine Aarhus University Aarhus Denmark; ^3^ Department of Autoimmunology and Biomarkers Statens Serum Institut Copenhagen Denmark; ^4^ Department of Ophthalmology Aarhus University Hospital Aarhus Denmark

**Keywords:** optic neuritis, optical coherence tomography, prognosis, steroid, treatment, visual function

## Abstract

**Objectives:**

Steroid treatment can accelerate visual recovery in patients with optic neuritis (ON), but it is unknown whether the timing of the start of treatment influences the outcome. The main purpose of this observational study was to assess the effect of early onset steroid treatment of ON on visual prognosis and retinal morphology.

**Methods:**

Forty‐nine patients with acute mild/moderate (*n* = 21) or severe (*n* = 28) ON, and an equal number of healthy controls were enrolled. Patients with severe ON either received early onset steroid treatment (initiated within 1 week of presentation with visual loss) (*n* = 9), late‐onset treatment (initiated after 1 week) (*n* = 13), or no treatment (*n* = 6). Visual function and retinal morphology was studied after 6 and 12 months.

**Results:**

All measures of visual function had improved after 6 months (*p *≤* *0.03) in the three groups with severe ON. This was not the case for Rayleigh match setting range (SR) in the nontreated group (*p *=* *0.24), or for SR (*p *=* *0.08) and latency to P100 of visual evoked potential (*p *=* *0.08) in the late‐onset treated group. After 12 months, further improvement occurred in the nontreated and late‐treated groups, but not in the early treated group. Macular retinal nerve fiber layer (mRNFL) and ganglion cell plus inner plexiform layer had decreased significantly (*p *≤* *0.001) in all three groups with severe ON after 6 months. After 12 months, only mRNFL had further significantly decreased and only in the late‐onset treated group (*p *=* *0.02).

**Conclusion:**

The beneficial effects of early onset steroid treatment of ON is limited to a few months whereas the long‐term prognosis is independent of the timing of treatment.

## 1 INTRODUCTION

Optic neuritis (ON) is characterized by subacute monocular visual loss, impaired color vision and periocular pain that worsens during eye movement (Galetta et al., 2015; Petzold & Plant, 2014). ON is often associated with demyelinating disorders such as multiple sclerosis (MS) and neuromyelitis optica spectrum disorder (NMOSD) (Jarius, Wildemann, & Paul, 2014; Oertel et al., 2017). The prognosis for visual recovery is generally good but contrast sensitivity and color vision are rarely normal, especially in patients with MS (Beck et al., 2004; Cole, Beck, Moke, Gal, & Long, 2000; Fleishman, Beck, Linares, & Klein, 1987). ON can cause axonal loss in the optic nerve, which can be visualized using optical coherence tomography (OCT) scanning and is seen as reduced thickness of the retinal nerve fiber (RNFL) and ganglion cell layer (GCL) (Costello et al., 2006, 2008; Syc et al., 2012).

Optic neuritis has several pathophysiological similarities with MS (Frohman et al., 2008), and treatment with high doses of methylprednisolone (steroid) has been shown to alleviate symptoms and accelerate recovery in both disorders (Beck et al., 1992; Brusaferri & Candelise, 2000; Miller et al., 2000). However, the time of initiation of treatment after symptom presentation may be important for the effect, for instance, the earlier the treatment is initiated there may be a chance for protecting visual function and retinal cell layers (Nakamura et al., 2010; Plant, Sibtain, & Thomas, 2011).

The objectives of this prospective study were to identify, examine, and follow‐up for a 1‐year period, new cases of acute ON in the Central Region of Denmark and to evaluate the effect of early onset steroid treatment in patients with severe ON.

## 2 MATERIALS AND METHODS

### 2.1 Study population

**Figure 1:**
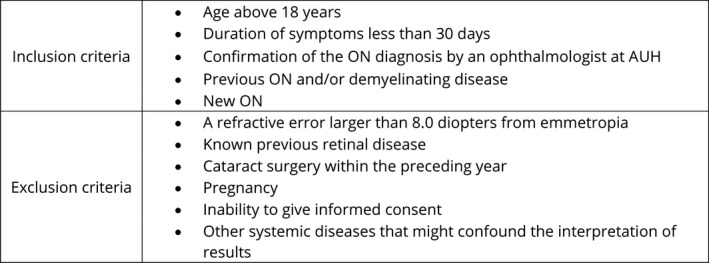
The inclusion and exclusion criteria of the study. AUH: Aarhus University Hospital; ON: optic neuritis

Ninety consecutive patients suspected of having ON that were referred to three neurological and two ophthalmological departments, and private ophthalmologists, in the Central Region of Denmark (population nearly 1.3 million citizens) between December 1st 2012 and May 31st 2014, were considered for inclusion in this study. Of these, 49 cases of ON fulfilling the inclusion and exclusion criteria shown in Figure 1 were included. An equal number of healthy controls recruited from hospital staff and their families was also included to obtain the best pairwise match with the patients with respect to sex (65% female), age (mean 34.9 ± 1.4 years), race (all were Caucasian, except for one patient who was mixed Caucasian/Inuit) and ocular refraction (*p *>* *0.34 for all comparisons).

The study was conducted according to the principles stated in the Declaration of Helsinki. It was approved by the regional ethics committee (1‐10‐72‐437‐12) and the Danish Health and Medicines Authority and was monitored by the GCP unit at Aarhus University Hospital (AUH). The study has been registered with EUDRACT (2012‐002628‐34). All participants provided written informed consent prior to study enrollment.

### 2.2 Neurological and paraclinical evaluation

At inclusion, all patients had a full neurological examination and a magnetic resonance imaging (MRI) of the brain and spinal cord. Previously healthy patients with abnormal MRI also had a lumbar puncture to measure the IgG‐index and rule out neuroinfection. The patients' blood samples were tested for the presence of anti‐aquaporin‐4 (AQP4) antibodies using the commercially available enzyme‐linked immunosorbent assay (ELISA) from RSR Limited (performed at Aarhus University). The blood samples were also tested for the presence of anti‐AQP4 antibodies and antibodies to myelin oligodendrocyte glycoprotein (MOG) using cell‐based indirect immunofluorescence assays (anti‐AQP4‐IIFT and anti‐MOG‐IIFT: Euroimmun, Luebeck, Germany). The anti‐AQP4‐IIFT and anti‐MOG‐IIFT were performed at Statens Serum Institute (SSI), Copenhagen, Denmark and results are recorded on a semiquantitative scale (negative, grey‐zone, weak positive, medium positive, or strong positive).

### 2.3 Ophthalmological tests

The following examinations were performed on both eyes of all patients:

Measurement of best corrected visual acuity (BCVA) using Early Treatment Diabetic Retinopathy Study (ETDRS) charts (Polaphor, Dortmund, Germany) in logMAR units and contrast sensitivity using Pelli‐Robson charts (Harlow, UK) in log units. Rayleigh match was obtained using the Tomey anomaloscope (Tomey All color anomaloscopie IF‐2, Nagoya, Japan), and the range of mixtures (the setting range) of red and green matching the reference yellow was noted as normal or abnormal. Computerized perimetry using the Humphrey Field Analyzer (Model 740i, Jena, Germany) and the 30‐2 full threshold test programme were used to note the overall field mean deviation (in dB). Visual evoked potentials (VEP) were measured using full field stimulation of a checkerboard pattern size corresponding to a visual angle of 19′ of arc. The stimulation was repeated twice with doubled pattern sizes each time. The mean latency to P100 (in ms) of the three recordings was calculated.

Optical coherence tomography (OCT) scanning was performed using the Heidelberg Spectralis HRA + OCT apparatus (Version 1.7.0.0, Heidelberg, Germany) with automatic real‐time (ART) function for image averaging. All OCT scans were performed with the same apparatus at the Department of Ophthalmology, AUH, and by the same operator (GHD). The examination room was dimly lit and the pupils were not dilated before scanning. The OCT scans were usually performed at the same day as testing of BCVA, contrast sensitivity, Rayleigh match and VEP. The perimetry was normally performed 1 day before the OCT scan.

Two types of scans were performed: A peripapillary ring scan to measure the peripapillary retinal nerve fiber layer (pRNFL) thickness (μm), and a macular volume scan to measure the mean thickness (μm) of the macular RNFL (mRNFL), the ganglion cell layer (GCL) in combination with the inner plexiform layer (IPL) termed GCIP, the inner nuclear layer (INL), the outer plexiform layer (OPL) in combination with the outer nuclear layer (ONL) denoted OPNL, and the photoreceptor layer (PRL). The peripapillary ring scan consisted of a manually placed ring measuring 3.4 mm in diameter around the optic nerve head, with eye tracker activated. Sixteen consecutive circular B‐scans (each consisting of 1,536 A‐scans) in high‐resolution mode was performed. The signal strength had to be >15 dB and 16 ≤  ART ≤ 100. The macular volume scan was centered on the fovea and had a scanning angle of 25° × 30°. The scan consisted of 61 vertical B‐scans (each consisting of 768 A‐scans) in high‐resolution mode. The signal strength had to be >15 dB and ART = 13. The pRNFL and macular intra‐retinal layers thickness was determined semiautomatically within a modified ETDRS circle (Early Treatment Diabetic Retinopathy Study Research Group, 1991) using the software Heidelberg Eye Explorer version 1.9.10.0 with viewing module 6.0.9, Heidelberg Engineering. All OCT examinations were repeated at least twice, checked for segmentation errors and corrected manually by the operator (GHD) if necessary. The best scan as assessed by OSCAR‐IB criteria (Tewarie et al., 2012) was selected. From all examinations the intereye difference in thickness from all retinal layers was calculated between the affected and the nonaffected eye. (Assessment of the intereye difference in for instance the GCIP layer identifies significantly more eyes with damage from ON than absolute values of thickness (Brandt et al., 2018).) The quantitative OCT data from this study are reported in line with the APOSTEL recommendations (Cruz‐Herranz et al., 2016).

In the healthy controls (HCs), measurement of BCVA and OCT scanning of both eyes (i.e., measurement of intereye retinal layer thickness difference) were performed. A blood sample was collected to measure anti‐AQP4 antibodies with ELISA (anti‐MOG antibodies were not tested in the HCs). The right eye was chosen as a comparison to the patient's affected eye.

The primary outcome measures were BCVA and thickness of the GCIP layer. Secondary outcome measures were contrast sensitivity, Rayleigh match, perimetry mean deviation, latency to P100, and thickness of pRNFL, mRNFL, INL, OPNL, and PRL layers.

### 2.4 Treatment

**Figure 2:**
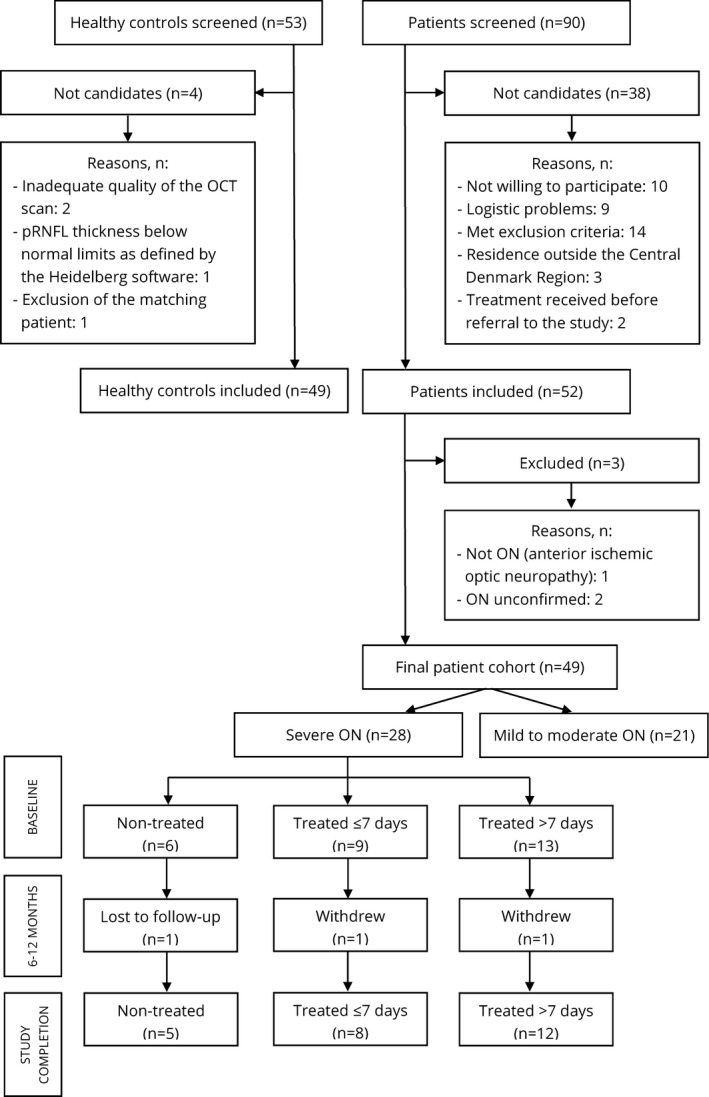
The patient selection. Among 90 screened patients, 52 were included. Three of these had to be excluded as the diagnosis could not be confirmed or was incorrect. This left a final patient cohort of 49 subjects. Among these, 28 had severe ON and were offered treatment. Six patients were not treated, the reasons being: patient's preference (*n* = 2); contraindications against methylprednisolone i.e., recent gastric surgery (*n* = 1), previous gastric ulcer (*n* = 2), lactation (*n* = 1). Nine patients were treated within 7 days and thirteen patients later than 7 days. One patient in each of the treated groups was either lost to follow‐up or withdrew after the 6 months visit. Among 53 screened healthy controls, 49 were included. OCT: optical coherence tomography; ON: optic neuritis; pRNFL: peripapillary retinal nerve fiber layer

Twenty‐eight patients with severe ON, defined as BCVA ≤ 0.5 decimal (0.30 logMAR) or BCVA >0.5 but severe amblyopia in the nonaffected eye (only one patient), were offered treatment with high‐dose intravenous methylprednisolone (Solu‐Medrol), 1 gram per day for 3–5 days (Figure 2). The 21 patients with mild/moderate ON were not offered treatment since the risk of side‐effects from the treatment was considered to be higher than the possible benefits.

To decrease the risk of side‐effects, oral treatment with a proton pump inhibitor (Pantoprazol 20 mg × 1 daily) and a 400 mg calcium and 19 μg (760 E) vitamin D combination (1 tablet × 2 daily) was added to the steroid treatment for the 3–5 days. If the patient reported severe side‐effects, the treatment would be terminated prematurely (*n* = 0).

### 2.5 Follow‐up

Follow‐up examinations of patients were performed at AUH after 6 and 12 months where all ophthalmological tests were repeated, except for perimetry at the 6 months visit and VEP at the 12 months visit. Three patients with severe ON left the study after the first follow‐up (Figure 2).

All the healthy controls completed a follow‐up visit after 5 months, consisting of BCVA measurement and OCT‐scanning.

### 2.6 Data analysis

Data were analyzed both in a modified intention‐to‐treat (ITT) population and the per‐protocol population. Secondary modified ITT and per‐protocol analyses excluded patients with previous and/or new ON in either eye, thereby avoiding confounding by repeated episodes of ON. In order to perform ITT analysis, the following three strategies were used: 1) Missing values due to not performed tests, were replaced by the mean of existing values in the total patient group. This was done for contrast sensitivity and Rayleigh match (one patient at baseline), perimetry (2 patients at 12 months) and macular OCT scans (six scans in four patients at baseline: unsuccessful scans bilaterally in two patients; in two patients the scan of the affected eye did not meet the OSCAR‐IB criteria). 2) Missing values due to poor visual function (giving “immeasurably” poor results) were replaced by the worst possible value for each test. For BCVA, only being able to see hand movements was set to +2.1 logMAR (two patients at baseline) and only light perception or complete blindness was set to +2.2 logMAR (four patients at baseline). For contrast sensitivity, the value was 0.00 log (10 patients at baseline). For Rayleigh match, the result was noted as “abnormal” (26 patients at baseline, three patients at 6 months, two patients at 12 months). For perimetry, the mean deviation was set to −35 dB (two patients at baseline). For VEP, 200 ms was chosen arbitrarily as latency to P100 (22 patients at baseline, six patients at 6 months) (the longest registered latency to P100 was 187 ms). 3) Carry‐over of values between 6 and 12 months. This was done for BCVA, contrast sensitivity and Rayleigh match (all tests in three patients, Rayleigh match in one patient), and for macular scans (11 scans in six patients: unsuccessful bilateral scan in two patients and in one patient's affected eye at 6 months; not performed bilateral scan in three other patients at 12 months) (Beck, Cleary, & Backlund, 1994; Costello et al., 2015; Henderson et al., 2010). Results presented in this article are from primary modified ITT analysis.

The patients with severe ON were subgrouped as to whether treatment was initiated within 1 week (denoted early onset) (*n* = 9) or later than 1 week (denoted late‐onset) (*n* = 13) of visual loss presentation, or not at all (*n* = 6). The 1‐week time limit was chosen as a “halfway cut‐off” since most patients with acute ON reach a nadir in visual function within 2 weeks after symptom onset (Beck, Cleary, & Backlund, 1994). This was suitable, since 19 of 22 (86.4%) patients received treatment within 2 weeks of symptom presentation (the last three were treated later because of a prolonged, gradual worsening of the visual function).

Continuous data were analyzed in a mixed model with nested effects and an unstructured covariance matrix for repeated measurement analysis of variance (ANOVA). An inspection of residuals and fitted values supported the validity of the model. Post hoc overall likelihood ratio tests and marginal Wald tests were calculated. Binary data were analyzed in a logistic regression with a robust cluster variance estimate to account for repeated measurements when appropriate.

Paired *t*‐test, unpaired *t*‐test, Wilcoxon test, Mann–Whitney *U* test, Kruskall–Wallis tests or one‐way ANOVA were used when appropriate for testing nonrepeated measurements (e.g., two paired or unpaired measurements or the mean of three group measurements).

Sample size in order to assess treatment effect of steroid, was estimated for at least 22 patients by using a reduction of 50% in expected pRNFL loss by early treatment (Costello et al., 2006), a power of 80%, a two‐sided significance level of 5% and an estimated standard deviation of 10.

Statistical significance was defined as *p *<* *0.05. All statistical analyses were performed using STATA12 (StataCorp LP, Texas, USA), http://scicrunch.org/resolver/SCR_012763.

## 3 RESULTS

### 3.1 Neurological and paraclinical evaluation

At inclusion all optic neuritis (ON) cases were demyelinating or idiopathic. Seven patients had previously been diagnosed with multiple sclerosis (MS), two with clinically isolated syndrome (CIS) and one with recurrent/bilateral ON (RON/BON). Two patients had previously had ON in the currently affected eye, and two patients had previously had ON in both eyes. The remaining 39 patients were previously healthy. None were seropositive for anti‐aquaporin‐4 antibodies. One patient was seropositive for antibodies to myelin oligodendrocyte glycoprotein, with medium intensity in the indirect immunofluorescence assay. This patient had RON/BON.

During the study period, 18 patients either previously healthy or known with CIS were diagnosed with MS and four with RON/BON. A total of four patients had a new ON in the same eye, and four had a new ON in the contralateral eye.

### 3.2 Ophthalmological tests

In all patients the best corrected visual acuity (BCVA) at baseline was worse in the affected eye (mean 0.61 ± SEM 0.12 logMAR) than in the nonaffected eye (−0.15 ± 0.01) and in the healthy controls (HCs) (−0.20 ± 0.01) (*p *<* *0.001 for all comparisons). After 6 months BCVA had improved in the affected eyes (−0.72 ± 0.11, *p *<* *0.001) whereas no further improvement had occurred after 12 months (−0.01 ± 0.01, *p *=* *0.31).

**Figure 3:**
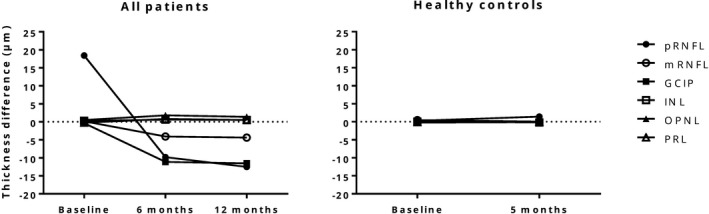
Longitudinal changes in intereye difference (i.e., affected eye – nonaffected eye) in mean thickness (μm) of retinal layers in the patients and healthy controls. The figure shows that at baseline, the intereye thickness difference in all retinal layers, except pRNFL, were similar between the patients and healthy controls. At follow‐up, however, clear changes appeared in the patient group, especially for GCIP, pRNFL and mRNFL, which decreased in thickness. This was in contrast with measurements in the healthy controls group where the retinal layers appeared stable. GCIP: ganglion cell layer in combination with inner plexiform layer; INL: inner nuclear layer; mRNFL: macular retinal nerve fiber layer; OPNL: outer plexiform layer in combination with outer nuclear layer; PRL: photoreceptor layer; pRNFL: peripapillary retinal nerve fiber layer

At baseline the patient group had a significantly larger intereye thickness difference in pRNFL than the HCs, but in none of the other retinal cell layers (Table S1). At 6 months, however, the patient group had a significantly larger intereye thickness difference in all retinal layers, compared to the HCs (Figure 3, Table S1).

Treated patients with severe ON had worse BCVA than nontreated patients with mild/moderate ON at all visits (*p *<* *0.01 for all comparisons). There was no significant difference in the intereye retinal layer thickness difference between treated patients with severe ON and nontreated patients with mild/moderate ON at baseline for any of the retinal layers. At 6 and 12 months, treated patients with severe ON had a significantly larger intereye thickness difference in the mRNFL and GCIP layers (but in none of the other layers), than patients with mild/moderate ON (mean group difference for mRNFL at 6 months: 2.13 μm; 95% CI: 0.32 to 3.94; *p *=* *0.021. Mean group difference for GCIP at 6 months: 5.37 μm; 95% CI: 0.43 to 10.32; *p *=* *0.033. Corresponding data for 12 months were similar. Figure S1.)

Table 1 shows that in the three groups of patients with severe ON all measures of visual function had improved after 6 months, except for the setting range (SR) in the nontreated group and both SR and latency to P100 in the late‐onset treated group. After 12 months the visual fields had improved significantly from baseline in all groups whereas improvement from the 6 months examination was observed for BCVA and SR in the nontreated group, for contrast sensitivity in the late‐onset group and for none of the parameters in the early onset treated group (Figure S2).

**Table 1:** Visual function in affected eyes among patients with severe ON

Visual function test	Nontreated (*n* = 6)			Treated ≤1 week (*n* = 9)			Treated >1 week (*n* = 13)		
	Mean (*SD*)			Mean (*SD*)			Mean (*SD*)		
	*p*			*p*			*p*		
	[95% CI]			[95% CI]			[95% CI]		
	Baseline^a^	6 months	12 months	Baseline^a^	6 months	12 months	Baseline^a^	6 months	12 months
BCVA, logMAR	1.50 (0.89)	−0.08 (0.12)	−0.13 (0.08)	0.98 (0.76)	−0.04 (0.23)	−0.04 (0.18)	1.05 (0.69)	−0.03 (0.23)	−0.05 (0.19)
	–	**<0.001**	**0.03**	–	**<0.001**	1.00	–	**<0.001**	0.25
	[0.30 to 2.20]	[−2.30 to −0.87]	[−0.09 to −0.01]	[0.30 to 2.20]	[−1.46 to −0.58]	[−0.09 to 0.09]	[0.10 to 2.10]	[−1.46 to −0.71]	[−0.06 to 0.02]
Contrast sensitivity, log	0.35 (0.41)	1.38 (0.24)	1.35 (0.25)	0.35 (0.34)	1.33 (0.29)	1.40 (0.20)	0.52 (0.50)	1.23 (0.33)	1.37 (0.26)
	–	**<0.001**	0.28	–	**<0.001**	0.06	–	**<0.001**	**0.004**
	[0.00 to 0.90]	[0.81 to 1.24]	[−0.07 to 0.02]	[0.00 to 0.75]	[0.79 to 1.17]	[−0.00 to 0.14]	[0.00 to 1.35]	[0.46 to 0.97]	[0.04 to 0.23]
Setting range, *n* abnormal (%)^b^	5 (83.33%)	3 (50.00%)	2 (33.33%)	9 (100.00%)	4 (44.44%)	3 (33.33%)	10 (76.92%)	3 (23.08%)	4 (30.77%)
	–	0.24	**0.04**	–	**0.01**	0.31	–	0.08	0.71
	–	[0.06 to 1.98]	[0.16 to 0.97]	–	[0.11 to 0.74]	[0.35 to 1.40]	–	[0.05 to 1.19]	[0.37 to 4.29]
Perimetry mean deviation, dB^c^	−23.50 (10.94)	–	−4.90 (3.62)	−23.95 (7.26)	–	−3.58 (3.18)	−22.11 (9.92)	–	−2.16 (1.34)
	–	–	**0.05**	–	–	**0.01**	–	–	**0.002**
	[−35.0 to −5.58]	–	[−11.59 to −1.66]	[−33.94 to −13.75]	–	[−10.30 to 0.03]	[−35.00 to −5.83]	–	[−4.42 to 0.21]
Latency to P100, ms^c^	184.61 (37.69)	129.02 (25.31)	–	200.00 (0.00)	151.33 (31.33)	–	169.62 (40.39)	148.54 (30.94)	–
	–	**0.03**	–	–	**0.01**	–	–	0.08	–
	[107.67 to 200.00]	[103.80 to 165.00]	–	[200.00 to 200.00]	[116.33 to 200.00]	–	[90.00 to 200.00]	[91.33 to 200.00]	–

*Notes*.

*p*‐values refer to the change from baseline to 6 months, and from 6 to 12 months. Significant *p*‐values are written in bold. Results are from ITT analysis.

BCVA: best corrected visual acuity; CI: confidence interval; dB: deciBel; ITT: intention‐to‐treat; log: logarithm; logMAR: logarithm of the Minimum Angle of Resolution; ms: millisecond; *SD*: standard deviation.

^a^Numbers in [] represent the range, min – max. ^b^p‐values and 95% CI are of the odds ratio. ^c^Numbers in [] represent range, min – max. No *p*‐values or 95% CI are stated because the data were analysed with nonparametric Wilcoxon signed‐rank test.

**Figure 4:**
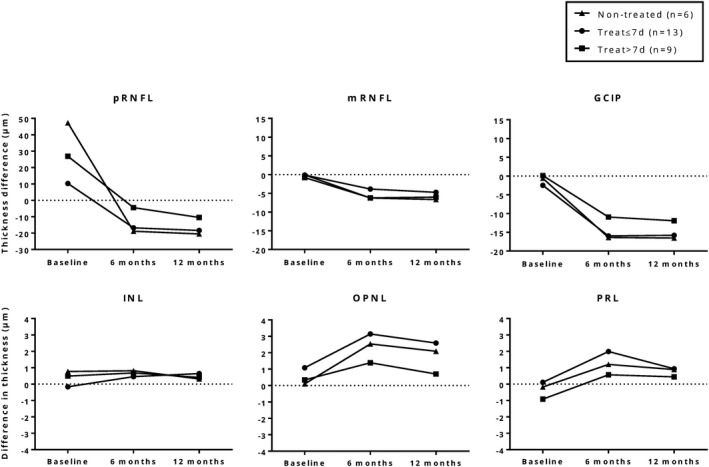
Mean thickness difference of retinal layers (i.e., affected eye – nonaffected eye) in the patients with severe ON. The largest reduction in retinal layer thickness could be seen at 6 and 12 months in the pRNFL and GCIP layer. However, there were no significant differences in the thickness of these layers between any of three patient groups. GCIP: ganglion cell layer in combination with inner plexiform layer; INL: inner nuclear layer; mRNFL: macular retinal nerve fiber layer; NonTreat: nontreated; OPNL: outer plexiform layer in combination with outer nuclear layer; PRL: photoreceptor layer; pRNFL: peripapillary retinal nerve fiber layer; Treat ≤ 7 d: treated within 7 days; Treat>7d: treated later than 7 days

Table 2 shows that after 6 months mRNFL and GCIP had decreased significantly in all three groups with severe ON. In the early onset and late‐onset treated groups PRL had increased (Figure 4). However, the intereye thickness difference of the PRL was significantly smaller in the late‐onset than in the early onset treated group (mean group difference −1.41 μm; 95% CI −2.48 to −0.35, *p *=* *0.01). From 6 to 12 months the mRNFL had decreased further in the late‐onset treated group, approaching the thickness difference in the other two groups. None of the other changes from 6 to 12 months were significant.

**Table 2:** Thickness of individual retinal layers and intereye thickness difference in the patients with severe ON

Retinal cell layer	Nontreated (*n* = 6)				Treated ≤1 week (*n* = 9)				Treated >1 week (*n* = 13)			
	Mean thickness, μm AE FE	Intereye thickness difference, μm Mean (*SD*) *p* [95% CI]			Mean thickness, μm AE FE	Intereye thickness difference, μm Mean (*SD*) *p* [95% CI]			Mean thickness, μm AE FE	Intereye thickness difference, μm Mean (*SD*) *p* [95% CI]		
	Baseline	Baseline	6 months	12 months	Baseline	Baseline	6 months	12 months	Baseline	Baseline	6 months	12 months
pRNFL	136.83 89.50	47.33 (104.75) – –	−18.83 (11.79) **0.01** [−114.76 to −17.58]	−20.50 (14.40) 0.82 [−15.66 to 12.32]	99.89 89.56	10.33 (13.17) – –	−16.78 (10.86) 0.18 [−66.78 to 2.56]	−18.33 (11.09) 0.79 [−12.98 to 9.87]	116.46 89.54	26.92 (36.15) – –	−4.46 (23.11) 0.06 [−64.39 to 1.63]	−10.46 (15.06) 0.22 [−15.50 to 3.50]
mRNFL	26.71 26.79	−0.08 (1.93) – –	−6.26 (3.99) **<0.001** [−9.17 to −3.19]	−6.65 (4.34) 0.50 [−1.52 to 0.74]	27.46 28.25	−0.78 (1.44) – –	−6.22 (3.72) **<0.001** [−7.88 to −3.00]	−6.02 (3.94) 0.68 [−0.73 to 1.12]	25.81 25.93	−0.12 (2.44) – –	−3.85 (3.92) **<0.001** [−5.75 to −1.69]	−4.74 (4.28) **0.02** [−1.67 to −0.13]
GCIP	71.53 72.14	−0.62 (1.83) – –	−16.41 (11.05) **<0.001** [−23.30 to −8.27]	−16.56 (11.24) 0.84 [−1.59 to 1.30]	70.59 73.09	−2.50 (2.03) – –	−16.00 (10.23) **<0.001** [−19.64 to −7.37]	−15.80 (10.24) 0.74 [−0.98 to 1.38]	69.50 69.38	0.12 (6.98) – –	−10.96 (9.84) **<0.001** [−16.18 to −5.97]	−11.94 (11.05) 0.05 [−1.96 to −0.00]
INL	34.54 33.77	0.77 (1.10) – –	0.81 (1.50) 0.95 [−1.38 to 1.47]	0.31 (1.59) 0.11 [−1.12 to 0.12]	34.76 34.93	−0.16 (1.10) – –	0.46 (1.47) 0.30 [−0.54 to 1.78]	0.64 (1.39) 0.47 [−0.32 to 0.69]	35.26 34.77	0.49 (2.61) – –	0.68 (1.88) 0.70 [−0.77 to 1.16]	0.41 (2.10) 0.20 [−0.69 to 0.15]
OPNL	98.96 98.86	0.10 (0.99) – –	2.54 (2.89) 0.20 [−1.31 to 6.18]	2.09 (2.31) 0.65 [−2.38 to 1.49]	98.89 97.80	1.08 (3.61) – –	3.15 (2.19) 0.19 [−0.99 to 5.12]	2.59 (1.63) 0.49 [−2.14 to 1.03]	101.12 100.77	0.35 (3.60) – –	1.39 (4.08) 0.42 [−1.50 to 3.59]	0.70 (4.69)0.30 [−2.01 to 0.62]
PRL	79.28 79.45	−0.17 (0.47) –	1.20 (1.20) 0.12 [−0.38 to 3.13]	0.89 (1.39) 0.63 [−1.58 to 0.96]	81.08 80.96	0.11 (1.59) – –	1.99 (1.36) **0.01** [0.44 to 3.31]	0.94 (1.05) 0.05 [−2.09 to −0.01]	80.99 81.91	−0.92 (2.52) – –	0.57 (0.89) **0.01** [0.30 to 2.68]	0.44 (0.98) 0.77 [−0.99 to 0.73]

*Notes*.

*p*‐values and 95% CI refer to changes in intereye differences from baseline to 6 months, and from 6 to 12 months. Significant *p*‐values are written in bold. Results are from ITT analysis.

AE: affected eye; CI: confidence interval; FE: fellow eye; GCIP: ganglion cell layer in combination with inner plexiform layer; INL: inner nuclear layer; ITT: intention‐to‐treat; mRNFL: macular retinal nerve fiber layer; OPNL: outer plexiform layer in combination with outer nuclear layer; PRL: photoreceptor layer; pRNFL: peripapillary retinal nerve fiber layer; *SD*: standard deviation; μm: micrometer.

To summarize, the inter eye thickness difference in all retinal layers at 6 and 12 months were similar (except for PRL at 6 months) in the three groups, irrespective of whether treatment was given or not, and irrespective of the timing of treatment.

## 4 DISCUSSION

In this observational and exploratory study, 49 patients with mild/moderate or severe acute optic neuritis (ON) were assessed and followed for 12 months with detailed registration of visual function and morphological retinal changes. Findings among the patients were compared to results from an equally large group of healthy controls (HCs). This study therefore contributes with important follow‐up data from optical coherence tomography (OCT) of HCs. Furthermore, this is the first prospective study to evaluate the effect of early versus late onset steroid treatment on visual function and retinal structure in patients with severe ON.

The present study is nicely in line with another recent Danish population‐based prospective study, in which 51 patients with acute ON were identified during a 2‐year period in the Region of Southern Denmark (population: almost 1 million citizens) (Soelberg et al., 2017). Therefore, with the identification of 49 patients with acute ON in the current study, the procedure for referral of patients with ON can be considered to have been nearly complete. The two Danish studies are also comparable when it comes to gender and age distribution. Furthermore, a similar number of patients were diagnosed with MS during the study period: 18 in the present study vs. 20 in the other study (Soelberg et al., 2017). In addition, no patients were seropositive for anti‐aquaporin‐4 antibodies, but both studies identified antibodies to myelin oligodendrocyte glycoprotein (MOG) in one patient with recurrent/bilateral ON (RON/BON) (Soelberg et al., 2017). The presence of anti‐MOG antibodies in patients with RON/BON is also in accordance with other studies (Chalmoukou et al., 2015; Pache et al., 2016).

The observed improvement of visual function from baseline to 6 and 12 months confirms the results from previous studies (Beck & Cleary, 1993; Beck et al., 1994; Jones & Brusa, 2003). The fact that the thickness of the peripapillar and macular retinal nerve fiber layer (pRNFL and mRNFL) and ganglion cell layer combined with the inner plexiform layer (GCIP) were reduced in the affected eye after 6 months, confirms that these cells are the primary cellular site of the disease (Al‐Louzi et al., 2015; Gabilondo et al., 2015; Henderson et al., 2010). The GCIP layer was the preferred outcome measure over the pRNFL and mRFNL, since the GCIP layer is unaffected by optic disc edema and therefore provides more precise information on the structural retinal changes caused by ON (Syc et al., 2012). The increased thickness of outer retinal layers after 6 months, followed by a slight thickness reduction of all retinal layers at 12 months may be due to involvement of the dentritical part of the retinal ganglion cells and a transitory reaction in the outer retina triggered by the inflammatory activity in the inner retina (Al‐Louzi et al., 2015; Gabilondo et al., 2015). Another possibility is that the observed increased thickness was caused by test–retest variability, even though the OSCAR‐IB criteria were rigorously followed (Oberwahrenbrock et al., 2015; Schippling et al., 2014; Tewarie et al., 2012).

Among patients with severe ON, an inverse relationship between pRNFL thickness (i.e., optic disc edema) and visual acuity was observed for nontreated patients, which is in agreement with previous findings (Beck et al., 1992; Henderson et al., 2010; Malik et al., 2014). The possible mechanism for the steroid treatment effect may involve a suppression of the inflammation associated with acute ON, which in turn may limit the neuronal damage and accelerate visual function recovery (Beck et al., 1992; Kapoor et al., 1998; Sellebjerg, Nielsen, Frederiksen, & Olesen, 1999). This is supported by the current study's observation that nontreated patients experienced an early reduction in the pRNFL thickness and a delayed improvement of visual acuity and color mixing. The increased thickness of the photoreceptor layer in the treated groups after 6 months confirms findings of swelling of the outer retinal layers in eyes treated with steroids (Gabilondo et al., 2015). This seemingly paradoxical reaction could not be related to changes in the inner retinal layers as also observed by others (Al‐Louzi et al., 2015) and requires further investigation.

The finding that patients who received early onset treatment showed a faster recovery but ended with the same final visual function and retinal damage as the patients who were not treated, is in accordance with the results of several studies (Beck & Cleary, 1993; Naismith et al., 2009; Sellebjerg et al., 1999). It would seem logical that the earlier the steroid treatment is initiated, the better the chances of a beneficial effect, but other studies showing this (Nakamura et al., 2010; Plant et al., 2011) may have been hampered by underpowering, heterogeneity of the background diagnoses of the patients and inconsistencies in follow‐up.

This study may contain some potential limitations: 1) The sample size was relatively small and only slightly more than half of the identified patients had severe ON and were included in the comparison of early versus late onset treatment. However, based on the sample size estimate a sufficient number of patients were included in the steroid treatment study, and the hypothesis that the timing of treatment initiation is important was rejected with a power of 80% when using our results from GCIP measurements. 2) The decision to treat and the timing of treatment was not randomized. However, a lack of selection bias is confirmed by the lack of significant differences in visual function and retinal morphology at baseline among the groups qualifying for treatment (Tables S2 and S3). The timing of treatment was a result of organizational factors unrelated to the disease severity. The relevant hospital departments and private ophthalmologists were continuously reminded to refer patients to study participation as quickly as possible to minimize the delay before any steroid treatment was initiated. It could be argued that early onset treatment should have been initiated even earlier, preferably within 3 days (Nakamura et al., 2010). However, in a clinical setting this is very difficult to achieve. The symptom presentation is not as acute as for instance in stroke and several factors influence the patient's decision to contact the health care system. 3) The inclusion of patients with both previously and newly diagnosed ON may have confounded the visual function outcomes and OCT measurements due to pre‐existing retinal damage (Beck et al., 2004; Cole et al., 2000; Trip et al., 2005). However, exclusion of patients with previous and/or new ON did not produce results conflicting with the primary modified ITT analyses. In addition, it has been shown that the rate of pRNFL thinning is similar in eyes from patients with MS, both when affected and unaffected by ON (Balk et al., 2016), which confirms that previous ON episodes are unlikely to have influenced the outcome. 4) The patients' vitamin D status was not routinely investigated in this study. It has been shown that vitamin D may be protective in acute ON (Burton et al., 2017). Vitamin D insufficiency is present in about 50% of the Danish population (Thuesen et al., 2012) and was, therefore, probably present in the current study population. It cannot be ruled out that the vitamin D status and supplement (19 μg (760 E) vitamin D given twice daily during the steroid treatment) had some effect on the outcome. However, there were no clear differences in either visual function or retinal morphological changes at baseline or during follow‐up between nontreated and treated patients with severe ON, which suggests that the vitamin D supplements did not affect the outcome. 5) Other tests, such as low contrast letter acuity (LCLA) and visual quality of life (QOL) could have added valuable information. Generally, testing of low contrast vision has been shown to be sensitive in detecting visual dysfunction in patients with MS (Baier et al., 2005). However, both LCLA measured with Sloan charts and contrast sensitivity measured with Pelli Robson charts, are sensitive tests (Balcer et al., 2003). Visual QOL (and low contrast vision) has been shown to be associated with thinning of pRNFL and GCIP in patients with MS (Sabadia et al., 2016).

In conclusion, early onset steroid treatment was found to accelerate visual improvement as compared to late‐onset treatment. Treatment as such (regardless of timing) accelerated improvement compared to nontreated patients. However, the final visual outcome was the same in all groups. The changes in individual retinal layers were similar in the early onset and late‐onset treated patients, and no significant beneficial effect could be found of steroid treatment compared to no treatment. OCT is a useful tool for monitoring structural changes in the retina during ON. The GCIP layer is recommended as the best retinal layer to monitor in future neuroprotective trials, since GCIP is unaffected by optic disc edema in the acute phase. Future research should focus on investigating the effect of other neuroprotective agents such as erythropoietin (Diem et al., 2016) for the treatment of acute ON, since the limited effect of steroids has now been well‐established.

## CONFLICTS OF INTEREST

None declared.
