# Supplementary figures and images for: Time to steroid treatment in severe acute optic neuritis
Source: Brain Behav. 2018 Jun 22;8(8):e01032. doi: 10.1002/brb3.1032 (PMC6085902; doi:10.1002/brb3.1032)

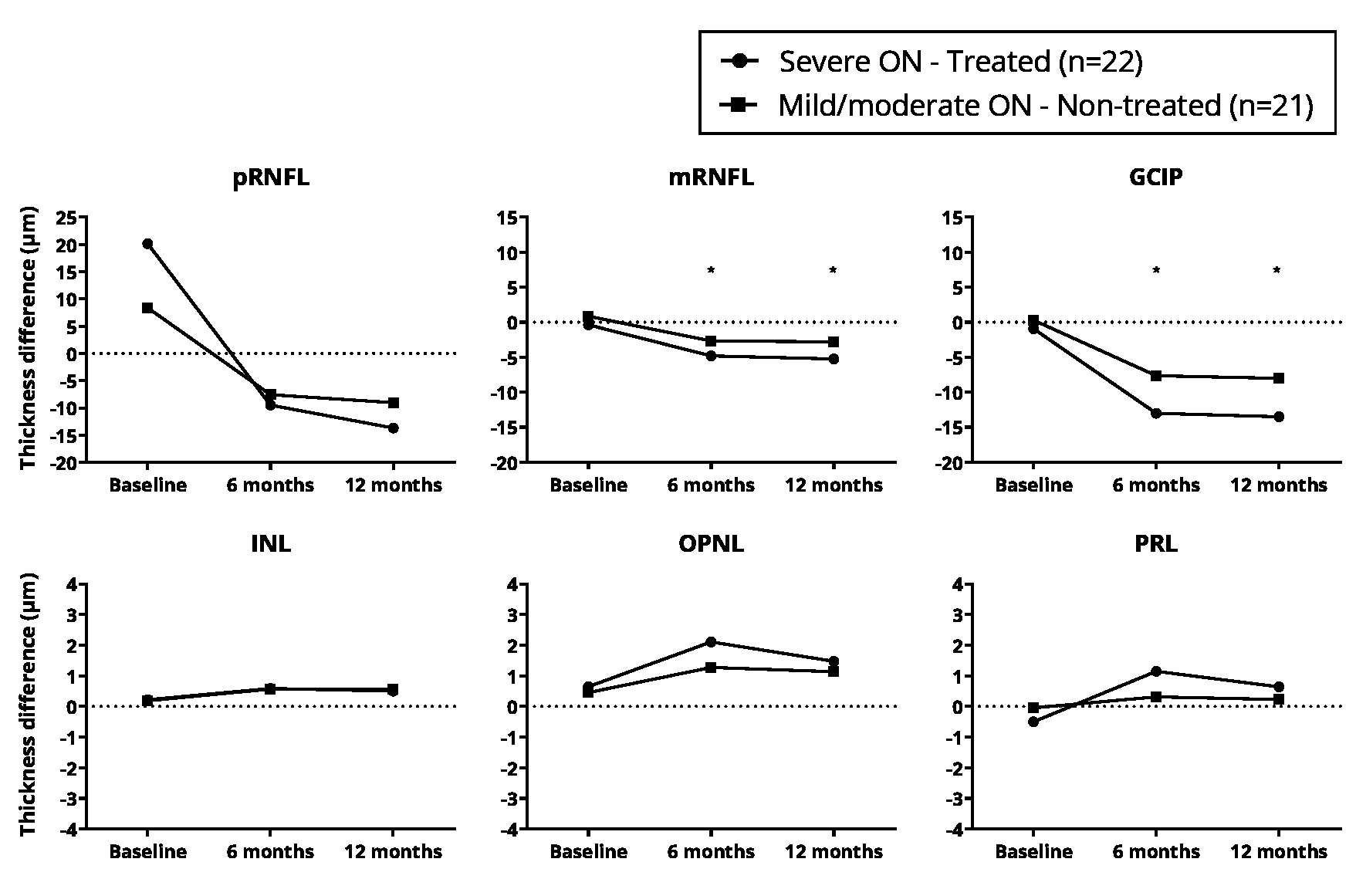

Supplement: Supplementary file 1 [file BRB3-8-e01032-s001.tif]

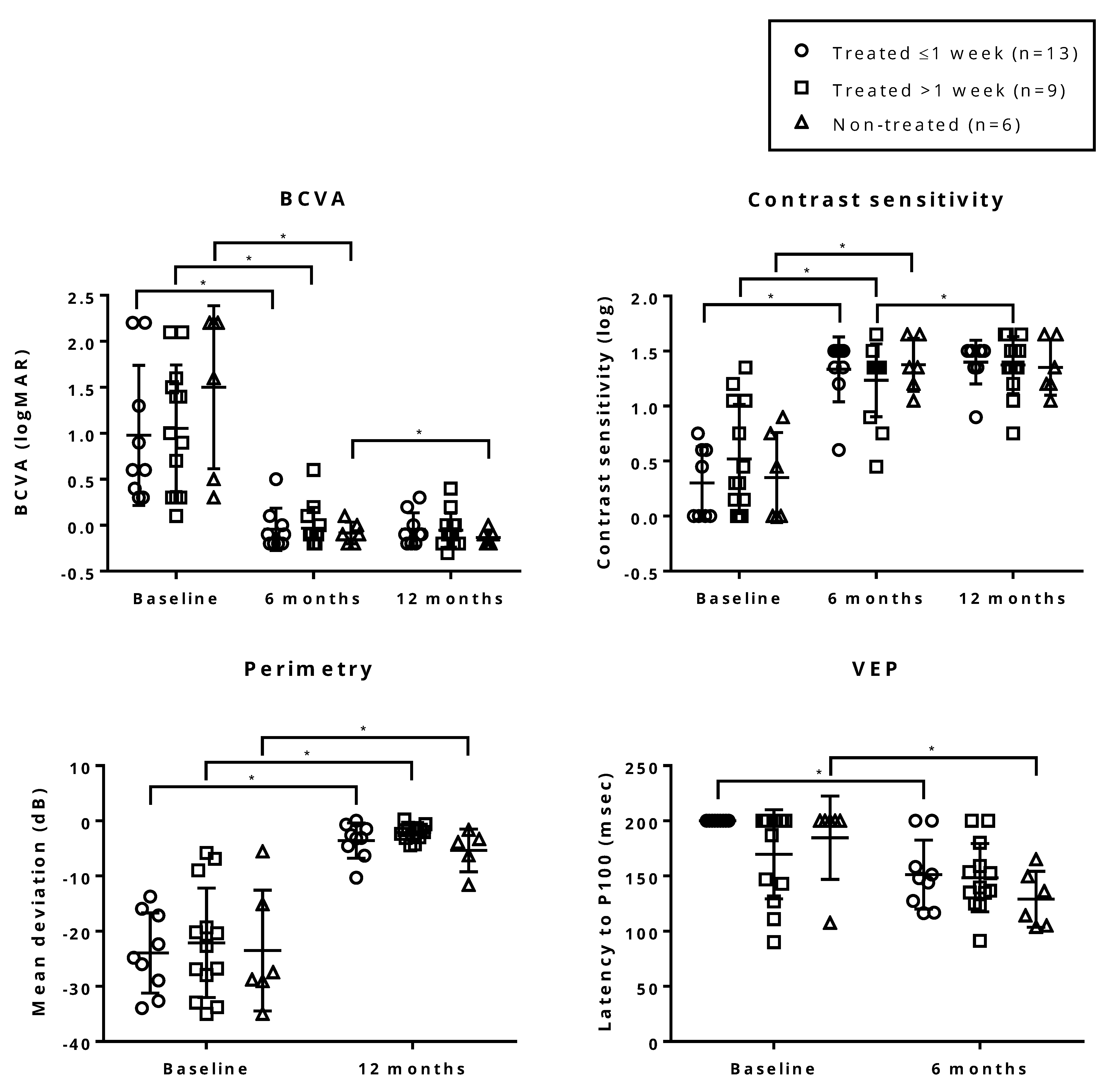

Supplement: Supplementary file 2 [file BRB3-8-e01032-s002.tif]
