# Supplementary material for: Time to steroid treatment in severe acute optic neuritis
Source: Brain Behav. 2018 Jun 22;8(8):e01032. doi: 10.1002/brb3.1032 (PMC6085902; doi:10.1002/brb3.1032)
Supplement: Supplementary file 3 [file BRB3-8-e01032-s003.docx]

**Table S1.** Inter-eye retinal layer thickness difference (i.e. affected eye – non-affected eye) for patients and healthy controls, as well as group comparison, at baseline and 6 months.

| **Retinal cell layer** | **Examination time** | **Patients**  **(n=49)** | **Healthy controls (n=49)** | **Group comparison,**  **patients vs. healthy controls** | | |
| --- | --- | --- | --- | --- | --- | --- |
|  |  | Inter-eye thickness difference, µm  Mean (SD) | | Inter-eye thickness difference, µm  Mean (SE) | p-value | 95% CI |
| pRNFL | Baseline | 10.83 (15.18)^a^ | 0.29 (2.31) | -10.54 (2.24) | **0.000** | -14.94 – -6.15 |
|  | 6 months | -9.11 (14.86)^a^ | 1.45 (2.26) | 10.56 (2.24) | **0.000** | 6.16 – 14.95 |
| mRNFL | Baseline | 0.17 (1.92) | 0.48 (1.29) | 0.30 (0.53) | 0.563 | -0.73 – 1.34 |
|  | 6 months | -4.08 (3.58) | 0.05 (1.49) | 4.14 (0.53) | **0.000** | 3.10 – 5.17 |
| GCIP | Baseline | -0.39 (4.69) | -0.11 (1.51) | 0.28 (0.69) | 0.687 | -1.08 – 1.64 |
|  | 6 months | -11.13 (9.28) | -0.07 (1.08) | 11.06 (1.33) | **0.000** | 8.45 – 13.67 |
| INL | Baseline | 0.28 (1.52) | 0.03 (0.68) | -0.25 (0.25) | 0.318 | -0.74 – 0.24 |
|  | 6 months | 0.61 (1.37) | -0.20 (0.69) | -0.81 (0.25) | **0.001** | -1.30 – -0.32 |
| OPNL | Baseline | 0.50 (2.52) | -0.21 (1.24) | -0.71 (0.53) | 0.182 | -1.76 – 0.33 |
|  | 6 months | 1.80 (2.76) | -0.11 (1.22) | -1.92 (0.53) | **0.000** | -2.96 – -0.87 |
| PRL | Baseline | -0.26 (1.64) | -0.11 (1.10) | 0.16 (0.28) | 0.586 | -0.40 – 0.71 |
|  | 6 months | 0.80 (1.38) | -0.06 (1.03) | -0.86 (0.28) | **0.003** | -1.42 – -0.30 |
