# Supplementary material for: Time to steroid treatment in severe acute optic neuritis
Source: Brain Behav. 2018 Jun 22;8(8):e01032. doi: 10.1002/brb3.1032 (PMC6085902; doi:10.1002/brb3.1032)
Supplement: Supplementary file 4 [file BRB3-8-e01032-s004.docx]

**Table S2.** Baseline visual tests of patients with severe ON.

| **Visual function test** | **Non-treated** (n=6)  Mean (SD) | **Treat≤7d** (n=9)  Mean (SD) | **Treat>7d** (n=13)  Mean (SD) | p-value |
| --- | --- | --- | --- | --- |
| BCVA, logMAR | 1.50 (0.86) | 0.98 (0.76) | 1.05 (0.69) | >0.05^a^ (RMA) |
| Contrast sensitivity, log | 0.35 (0.41) | 0.35 (0.34) | 0.52 (0.50) | >0.05^a^ (RMA) |
| Setting range, n eyes abnormal (%) | 5  (83.33%) | 9  (100.00%) | 10  (76.92%) | >0.05^a^ (LRRC) |
| Perimetry mean deviation, dB | -23.50 (10.94) | -23.95 (7.26) | -22.11 (9.92) | >0.05^a^ (1W) |
| Latency to P100, msec | 184.61 (37.69) | 200.00 (0.00) | 169.62 (40.39) | 0.06  (KW) |
