# Supplementary material for: Time to steroid treatment in severe acute optic neuritis
Source: Brain Behav. 2018 Jun 22;8(8):e01032. doi: 10.1002/brb3.1032 (PMC6085902; doi:10.1002/brb3.1032)
Supplement: Supplementary file 5 [file BRB3-8-e01032-s005.docx]

**Table S3.** Pairwise comparison of inter-eye retinal thickness difference at baseline between the groups with severe ON.

|  | **Non-treated vs. Treat≤7d** | | | **Non-treated vs. Treat>7d** | | | **Treat≤7d vs. Treat>7d** | | |
| --- | --- | --- | --- | --- | --- | --- | --- | --- | --- |
| **Retinal cell layer** | Mean inter-eye thickness difference, µm (SE) | p-value | 95% CI | Mean inter-eye thickness difference, µm (SE) | p-value | 95% CI | Mean inter-eye thickness difference, µm (SE) | p-value | 95% CI |
| pRNFL | 37.00 (28.73) | 0.198 | -19.30 – 93.30 | 20.41 (26.90) | 0.448 | -32.31 – 73.13 | 16.59 (23.63) | 0.483 | -29.73 – 62.91 |
| mRNFL | 0.70 (1.10) | 0.524 | -1.46 – 2.86 | 0.04 (1.03) | 0.968 | -1.98 – 2.06 | 0.66 (0.91) | 0.466 | -1.12 – 2.44 |
| GCIP | 1.88 (2.83) | 0.508 | -3.68 – 7.44 | -0.73 (2.66) | 0.782 | -5.94 – 4.47 | 2.61 (2.34) | 0.263 | -1.96 – 7.19 |
| INL | 0.93 (1.12) | 0.405 | -1.26 – 3.12 | 0.28 (1.05) | 0.791 | -1.77 – 2.33 | 0.65 (0.92) | 0.477 | -1.15 – 2.46 |
| OPNL | -0.98 (1.94) | 0.612 | -4.78 – 2.81 | -0.24 (1.81) | 0.893 | -3.80 – 3.31 | -0.74 (1.59) | 0.643 | -3.86 – 2.38 |
| PRL | -0.29 (1.03) | 0.779 | -2.30 – 1.72 | 0.74 (0.96) | 0.438 | -1.14 – 2.63 | -1.03 (0.84) | 0.221 | -2.68 – 0.62 |
